# Cross‐species systems biology target prioritization leads to the development of antisense oligonucleotides as a potential therapeutic approach for progressive supranuclear palsy

**DOI:** 10.1002/alz.090392

**Published:** 2025-01-03

**Authors:** Yuhao Min, Xue Wang, Jeremiah Bergman, Katie D Sotelo, Joseph S. Reddy, Zachary Quicksall, Thuy Nguyen, Adriana O Mitchell, Dennis W. Dickson, Keiji Kawatani, Yasuteru Inoue, Takahisa Kanekiyo, Mariet Allen, Ozkan Is, Nilüfer Ertekin‐Taner

**Affiliations:** ^1^ Mayo Clinic, Jacksonville, FL USA; ^2^ Department of Neuroscience, Mayo Clinic, Jacksonville, FL USA

## Abstract

**Background:**

Progressive supranuclear palsy (PSP) is a devastating primary tauopathy with rapid progression to death. Although several therapies currently in the development pipeline show promising safety profiles and robust target engagement, few demonstrated significant efficacy in patients, underscoring the need to interrogate additional targets with novel therapeutic modalities to expand the potential therapeutic arsenal. To diversify the therapeutic avenues for PSP and related tauopathies (e.g. Alzheimer’s disease), we systematically integrated multi‐omics data from human brains of PSP and control donors with cross‐species validation to nominate high‐confidence therapeutic targets. We plan to translate our findings into safe and effective treatments for PSP using antisense oligonucleotides (ASO).

**Method:**

We analyzed brain gene expression profiles in PSP and control individuals at bulk tissue (N = 408) and single‐cell levels (snRNAseq, N = 36). Cell‐type‐specific expression perturbations were systematically prioritized using a cross‐species validation paradigm, including rTG4510 tau mice and tau‐overexpressing *Drosophila* models. We conducted *in vitro* screening of ASO candidates against our prioritized target genes. ASO efficacy and toxicity were measured. RNAseq experiments will be performed to interrogate off‐target effects. The safety and efficacy of the lead ASOs will be assessed in PSP‐patient‐derived iPSC models.

**Result:**

We previously reported discovery of novel genes with significant differential expression in PSP brains, characterized their brain cell‐specificity and validated them using snRNAseq data. We prioritized 21 genes using gene expression data from a mouse tauopathy model. Validation of these 21 high‐priority genes using a *Drosophila* tau model nominated astrocytic *STOM*, *KANK2*, and *DDR2* as potential therapeutic targets for PSP. Knocking down their expression in *Drosophila* significantly rescues tau‐mediated neurodegenerative pathology. *In vitro* screening identified ASO leads that reduced the target expressions at mRNA and protein levels with low cellular toxicity.

**Conclusion:**

We developed a systems biology pipeline and nominated *STOM*, *KANK2*, and *DDR2* as candidate gene targets for PSP. We identified ASOs that modulate these targets’ expression without cellular toxicity, suggesting they may be suitable as potential therapeutic candidates. Importantly, the shared pathophysiology and molecular aberrations between PSP and other tauopathies, such as Alzheimer’s disease, suggests that such therapeutic candidates may be repurposed for multiple neurodegenerative diseases, further accelerating, and streamlining the therapeutic pipeline.

